# Deletion of a Malaria Invasion Gene Reduces Death and Anemia, in Model Hosts

**DOI:** 10.1371/journal.pone.0025477

**Published:** 2011-09-28

**Authors:** Noé D. Gómez, Innocent Safeukui, Aanuoluwa A. Adelani, Rita Tewari, Janardan K. Reddy, Sam Rao, Anthony Holder, Pierre Buffet, Narla Mohandas, Kasturi Haldar

**Affiliations:** 1 Center for Rare and Neglected Diseases, University of Notre Dame, Notre Dame, Indiana, United States of America; 2 Feinberg School of Medicine, Department of Pathology, Northwestern University, Chicago, Illinois, United States of America; 3 Centre for Genetics and Genomics, School of Biology, University of Nottingham, Nottingham, United Kingdom; 4 Division of Parasitology, MRC National Institute for Medical Research, London, United Kingdom; 5 Inserm-UPMC (Paris 6 University) Unité Mixte de Recherche s945, Paris, France; 6 New York Blood Center, New York, New York, United States of America; Bernhard Nocht Institute for Tropical Medicine, Germany

## Abstract

Malaria parasites induce complex cellular and clinical phenotypes, including anemia, cerebral malaria and death in a wide range of mammalian hosts. Host genes and parasite ‘toxins’ have been implicated in malarial disease, but the contribution of parasite genes remains to be fully defined. Here we assess disease in BALB/c mice and Wistar rats infected by the rodent malaria parasite *Plasmodium berghei* with a gene knock out for merozoite surface protein (MSP) 7. MSP7 is not essential for infection but in *P. falciparum*, it enhances erythrocyte invasion by 20%. *In vivo*, as compared to wild type, the *P. berghei Δmsp7* mutant is associated with an abrogation of death and a decrease from 3% to 2% in peak, circulating parasitemia. The *Δmsp7* mutant is also associated with less anemia and modest increase in the size of follicles in the spleen. Together these data show that deletion of a single parasite invasion ligand modulates blood stage disease, as measured by death and anemia. This work is the first to assess the contribution of a gene present in all plasmodial species in severe disease.

## Introduction

Malaria is one of the world's major health problems. It results from infection by parasites belonging to the genus *Plasmodium*. *Plasmodium falciparum* causes the most virulent form of human malaria and kills close to one million children annually. The asexual blood stage parasite infects the red blood cell (RBC) and is responsible for all of the symptoms and pathology associated with malaria. Uncomplicated malaria consists of unspecific fever, chills, and diffuse pain. Severe malaria includes multiple additional pathologies including anemia, respiratory distress, lactic acidosis and cerebral malaria [1], [2] and greatly increases the risk of fatal outcome.

Over the past decade there has been significant progress in the identification of parasite ligands and host receptors involved in the establishment of acute infection. Host genes associated with severe disease have also been examined [3]. Further, parasite factors such as the black pigment of malaria hemozoin as well as glypiated lipids that anchor parasite proteins to membranes have been implicated in causation of severe malaria [4], [5]. However the contribution of parasite genes to severe disease remains poorly understood. This requires utilization of genetically modified parasites as well as suitable host disease models. There is recognition that animal models can provide useful insights into pathologies of severe malaria including human disease [6], [7], [8] but there is a paucity of validated disease models in which modified parasites can be tested for the manifestation of disease *in vivo*. Anemia has been studied in murine models, but the parasite strains used are genetically not tractable [9], [10]. Moreover, while it is possible to delete genes in several species of *Plasmodium*, the process is not as rapid as in model pathogenic bacteria and yeast [11], [12], [13], [14], [15]. Thus it is necessary to prioritize genes to target in the parasite as well as the disease models in which to test them.

Parasite encoded proteins that can remodel RBC have been proposed to play a role in anemia, the most prevalent and inflammatory pathology of malaria. Candidate proteins may reside on the surface and in apical organelles of invading parasites and be involved in entry, or bear a ‘host-targeting’ (HT) or plasmodial export element (PEXEL) that are exported to the erythrocyte [16], [17]. Many are released into plasma either as part of their physiological processing or upon rupture of the infected erythrocyte, and especially at high concentration and may adhere to uninfected erythrocytes and trigger their removal from circulation. Alternatively, these parasite proteins may be transferred to the RBC surface upon abortive invasion [18]. Removal of uninfected erythrocytes from circulation is a major contributing factor in anemia [19], [20]. Since anemia is caused by all *Plasmodium* species, and is often associated with partial immunity, erythrocyte-adhesive proteins that induce anemia are expected to be conserved through the genus.

On the basis of these criteria, we selected merozoite surface protein 7 (MSP7), which in *P. falciparum*, forms a complex with MSP1 and MSP6 [21]. This complex has been proposed as a vaccine target. Antibodies to MSP7 are detected in human immune serum [22]. *P. falciparum msp*7 can be deleted with a consequent 20% reduction in merozoite invasion of erythrocytes [23]. In *P. falciparum, msp7* is the prototypic representative of six related genes, in a tandem array on chromosome 13. This family is expanded in *P. vivax* to 11 genes [24]. The murine malaria parasites *P. berghei* and *P. yoelii* have only three *msp7-*like genes at the corresponding genomic location. It has been suggested that *P. yoelii* MSP7 proteins are a target of host immune responses [25]. In *P. berghei*, deletion of the middle gene (designated as *msp7*) was shown to delay initial parasite growth *in vivo*, although no difference in growth rate was observed later during infection [26]. This result emphasizes the possibility that MSP7 may play a relatively minor role in erythrocyte invasion in a susceptible host, and led us to consider testing its contribution to *P. berghei*-induced disease.

## Methods

### Ethics Statement

This study was carried out in strict accordance with the recommendations in the Guide for the Care and Use of Laboratory Animals of the National Institutes of Health. Northwestern (A3283-01) and the University of Notre Dame (A3093-01) are credited through the Animal Welfare Assurance. All procedures involving animals were reviewed and approved by the Northwestern University (Protocol #2006-0935) and the University of Notre Dame (Protocol #11-070) IACUC committees. All efforts were made to minimize suffering of the animals.

### Disruption of *msp7* in *P. berghei* and confirmation of Δ*msp7*

Plasmid pDHΔMSP7 [26] was used to transfect *P. berghei* ANKA schizonts as previously described [27]. The resultant gene knockout parasites ***–*** *Δmsp7* were cloned and confirmed by Southern blotting to have the same deletion as in the original line created previously [26] (Figures S1A–S1C).

### Growth of wild type versus Δ*msp7* mutant parasites

To reexamine the effect of *msp*7 disruption on parasite growth *in vivo*, challenge inocula (1000 infected RBCs) from *P. berghei* ANKA wild type (WT) and *Δmsp7* lines were injected intravenously (i.v.) into groups of BALB/c mice that were 6–8 weeks old. Forty-eight hours post-infection (p.i.) and every other following day for the next 10 days, Giemsa-stained thin blood films were made and examined for each mouse. Parasitemia is expressed as a percentage of RBC that are infected, based on counting of at least 2000 RBCs.

### Generation of semi-immune mice for study of chronic infections

Two separate cohorts of 6–8 week old BALB/c mice (WT and Δ*msp7* groups) were infected intraperitoneally (i.p.) with 10^4^ infected RBCs and then treated at day 6 p.i. with chloroquine (10 mg/kg) and pyrimethamine (10 mg/kg), both administered i.p. daily for 5 days as previously described [28]. During subsequent rounds of infection mice were rested for 2 weeks prior to re-challenge with 10^4^ cells of either wild type or *Δmsp7 P. berghei* ANKA, then monitored and drug treated before any of the mice in the cohort reached 5% parasitemia. Mice underwent 4 or 5 cycles of infection and drug cure before receiving a final challenge of 10^4^ cells of their respective parasite line without treatment.

### Infection in an aged mouse model

20 to 24 week (5–6 month)-old male BALB/c mice were injected i.p with10^4^ or i.v. with 10^3^ *P. berghei* ANKA (Malaria Research and Reference Reagent Resource Center [MR4] Parasite # MRA-311). Forty-eight hours post-infection (p.i.) and every other following day for the next 10 days, Giemsa-stained thin blood films were made and examined for each mouse. Parasitemia is expressed as a percentage of infected cells, based on counting of at least 500 RBCs. Where indicated, hemoglobin (Hb) was measured in a 96-well plate by absorbance at 540 nm using 2 µl of tail vein blood in 0.5 mL Drabkin's Reagent (Sigma, St. Louis, MO), and is expressed as percent of baseline.

### Infection in an aged rat model

15 week-old Wistar rats were injected i.p. with 10^6^ *P. berghei* ANKA (Malaria Research and Reference Reagent Resource Center [MR4] Parasite # MRA-311) infected RBCs, respectively. Parasite levels were monitored every 2 days by Giemsa-stained thin blood film and expressed as a percentage, counting more than 500 RBCs. Where indicated, hemoglobin (Hb) was measured in a 96-well plate by absorbance at 540 nm using 2 µl of tail vein blood in 0.5 mL Drabkin's Reagent (Sigma, St. Louis, MO), and is expressed as percent of baseline. Two independent mutants of *msp7* were tested, one generated previously [26] and the second as described above.

### Multi-Analyte Analysis

Whole blood was obtained by terminal cardiac puncture in the presence of an anticoagulant and then centrifuged to obtain plasma. A Luminex-based bead array (RodentMAP version 2.0; Rules Based Medicine, Inc., Austin, TX, USA) was used to simultaneously assess the level of 59 unique analytes. Plasma from rats at days 8 and 10 p.i. were analyzed.

### Histopathological analysis

Lung, liver and spleen were harvested from infected (with wild type or *Δmsp7 P. berghei*) and uninfected Wistar rats. Harvested organs were washed with PBS to remove blood, placed into fixative (10% neutral buffered formalin) for 15 minutes, and then cut into smaller portions. Cut portions were transferred to and fully immersed in fixative at 4°C for 1 hour, and then left at 4°C overnight in fresh fixative. Samples were then processed, paraffin embedded, and thin sections (3–4 µm) produced with a microtome, and stained with Hematoxylin and Eosin at AML Labs Inc. (Baltimore, MD, USA). Spleen follicle size was determined by using a squared grid over images of spleens and calculating the number of grid squares (1 cm^2^) covering the follicle.

### Statistical Analysis

All graphs show the standard error of the mean (SEM). Differences in parasitemia, follicle size, and anemia were measured statistically using the Student's t-test.

## Results

### Effect of *msp7* deletion in acute and chronic infection by *P. berghei* in mice

**Figure 1:**
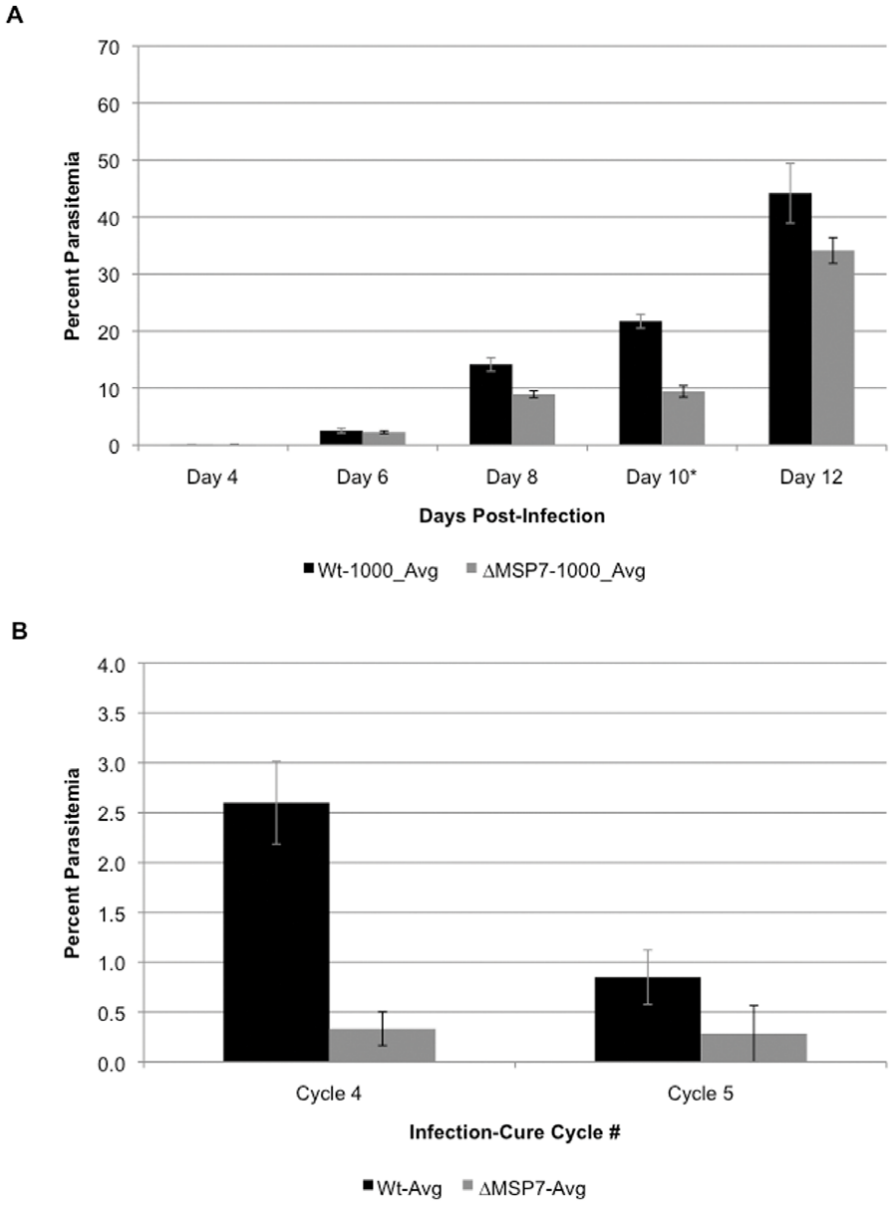
Analysis of wild type and Δ*msp7* mutant parasites in acute infection or semi-immune mouse models. (A) For acute infection, all 6–8 week old mice were given inocula of 1000 infected RBCs of the respective parasite lines and monitored every other day by thin blood film. *Day 10 is based on data from 5 mice, while all other days are based on data from 10 mice (SEM shown). (B) Semi-immune mouse model showing the average parasitemia of wild type and Δ*msp7 P. berghei* infected mice immediately before drug cure in cycles 4 and 5 of the infection-cure protocol (SEM shown).

Prior studies suggested that in acute infection in mice, *Δmsp7* mutants display small differences in parasitemia compared to their wild type counterparts [26]. We made a second *Δmsp7* mutant parasite line, which was confirmed to have *msp7* missing by integration PCR and Southern blot analysis (Figure S1A–S1C). A slower increase of parasitemia was observed in animals infected with the *Δmsp7* mutant parasite over the first 8 to 10 days p.i. This average difference in parasitemia was markedly reduced by day 12 p.i. when animals in both groups displayed 30–45% parasitemia (Figure 1A). In prior studies Tewari et al., [26] reported 17% parasitemia on day 10, which was higher than the 10% reported here. However at day 12, Tewari reported 30% parasitemia, which corresponds well (within statistical significance) to the data in our current studies. The reasons for the discrepancies on day 10 are unclear. Possible explanations could be that, the mice in the present study may be slightly younger or older and that there were small differences in the absolute number injected parasites or the reticulocytes on Day 10. But over all the data confirm earlier studies [26] showing that deletion of *msp7* gene does not have a major effect on acute infection in 6–8 week old mice. We were next interested to assess the mutants in murine models of chronic infection. Evans et al. [28] proposed a semi-immune mouse model where the animals are subjected to repeated cycles of infection and cure. In this model we found that after three cycles, mice injected with the *Δmsp7* parasite showed significantly lower parasitemias relative to wild type-infected mice in the fourth cycle (Figure 1B). Upon the fifth challenge, both cohorts controlled infection almost equally well with an average parasitemia under 5% (Figure 1B). These data suggest that the rise of parasitemia of the *Δmsp7* mutant may be controlled more rapidly upon re-infection relative to the wild type parasite, even though the effect of MSP7 deletion is less evident in acute infection. However, since each cycle of infection and cure takes a month, by the fourth cycle (when the mutant parasite is attenuated), the animal is aged approximately 5–6 months. Thus age-dependent differences in mouse response to infection, may have also contributed to the relatively attenuated phenotype of *msp7* mutants seen in Figure 1B.

To test the effect of age, mice 5–6 months old were injected i.p. with 10^4^ wild type and *Δmsp7* mutants. As shown in Figure S2A, infection with wild type parasites resulted in a rapidly rising parasitemia and a high death rate with parasitemia reaching ∼42% at day 8. The *msp7* mutants induced a slower rise in parasitemia during the first ten days. Subsequently however, parasitemias approached 75%. Further, time to death was significantly increased in mice infected with *msp7* mutants (Figure S2B) even though they sustained higher parasitemias. Differences in hemoglobin levels could be explained by parasitemia (Figure S2A) and thus death cannot be ascribed to anemia per se. These data indicate that the age of the mouse could have a profound influence on the survival of animals infected with both wild type and *msp7*-deleted parasites. Thus models of chronic infection initiated in animals at 6 to 8 weeks of age but where partial immunity develops after a series of intraperitoneal injections at ∼5 months [28], may be compromised by prominent age dependent effects. In these models it is difficult to assess immunity obtained by antigen exposure alone.

To directly compare the effects of age on acute infection shown in Figure 1, we injected 10^3^ parasites i.v. into aged (5–6 month old) mice (Figure S3A). Here the mutants show a delay in growth but more prominently 50% of animals infected with wild type parasites die by day 12 (Figure S3B), while the mutants achieve same levels of death by day 21. In contrast in 6–8 week old mice, injected i.v. with the same parasite dose, 50% death was seen at day 16, for both wild type and mutant infections.

These data suggest that death in aged (5–6 month old) mice infected with wild type parasites may not be due to parasitemia alone, but may rather reflect additional pathogenic processes. We found elevated levels of multiple inflammatory cytokines in plasma taken from infections with wild type relative to mutant parasite (Figure S4), despite the fact that parasite burdens were consistently greater in animals infected with the mutants. However since parasite burdens were significantly different between the two groups, the extent to which these differences contribute to altered cytokine responses are unclear.

### Effects of deletion of *msp7* on death and anemia induced by *P. berghei* parasites in aged (15 week old) Wistar rat

**Figure 2:**
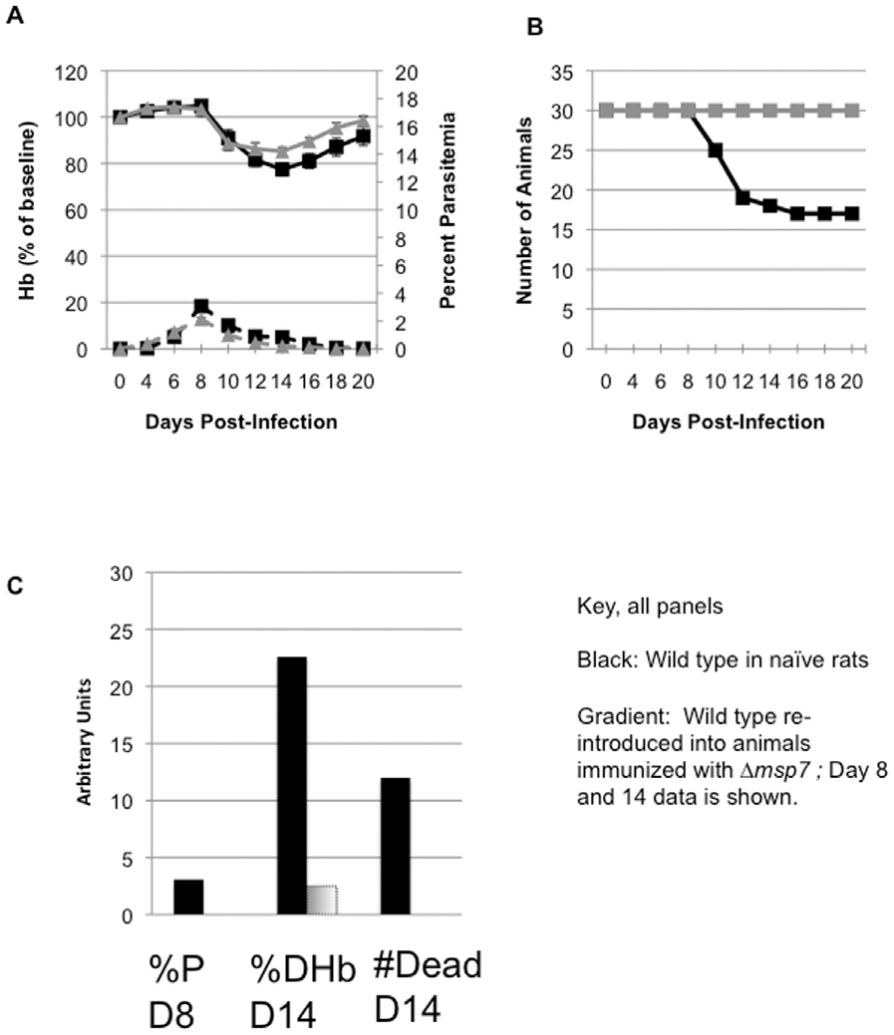
Aged rat model for anemia and death. (A) SMA model for aged rats, showing average percent of baseline Hb and average parasitemia. (B) Number of rats that survived in the aged rat model. The data are from three experiments, N = 30 rats. (C) Comparison of percent parasitemia (%P), percent hemoglobin drop (%Hb), and death (#Death) in naïve rats infected with wild type parasites (black bars) and rats previously infected with Δ*msp7* parasites and then re-infected with wild type parasites (gradient bars).

To overcome high parasitemia- and age-dependent changes during infection, we analyzed the effect of *msp7* deletion in the aged (15 week old) Wistar rat model. In this model, animals have previously been shown to mount an immune response to control *P. berghei* infection at low parasitemia and to manifest anemia [28]. Hence the rats were challenged with 10^6^ wild type parasites and then monitored for parasitemia as well as circulating hemoglobin level (Hb). As shown in Figure 2A, in wild type infections, all of the animals showed a low, self-resolving parasitemia. The kinetics of infection showed very little inter-individual variation. Peak parasitemias averaging ∼3.0% were seen largely around day 8 post-infection and were resolved by day 18. The drop in Hb levels started after day 8 with a nadir of about 78% of baseline by day 14. In addition, we observed death in infected rats starting after the first day of hemoglobin reduction (day 10). Of the 30 rats infected with wild type *P. berghei*, 13 died between days 10 and 16 post-infection (Figure 2B).

When animals were infected with *msp7*-deleted parasites the average peak parasitemia was 2% as compared to 3% induced by wild type infection (Figure 2A). Influx of reticulocytes was seen after peak parasitemias were controlled, i.e. after day 10 (not shown) and thus infection of reticulocytes was limited. The data from two independently generated mutants were very similar, confirming that the observed phenotypes were due to disruption of the *msp7* gene, and are combined here. The most striking phenotype was death, which was observed in none of the *msp7*-mutant infected rats even after up to 20 days of infection. This is in contrast to a 43% mortality rate in wild type infections (Figure 2B). In addition, the average drop in Hb levels seen in animals infected with the mutants was significantly less than that observed in rats infected with wild type parasites (14%, versus 22%, p<0.05, Figure 2A). By day 24, hemoglobin levels in both wild type and *msp7* mutant-infected animals were restored to normal levels (Figure 2A), consistent with the fact that in this model there is no significant impairment of erythropoiesis [28].

We also examined the outcome of infection with wild type parasites in animals that had previously recovered from infection with the *Δmsp7* parasites. No detectable parasitemia was observed in these animals (Figure 2C), hemoglobin levels remain unchanged, and no deaths were observed. These data, when taken in conjunction with the results shown in Figure 2A and B suggest that manifestation of both host death and anemia may be intimately linked to both malarial infection *and* the presence of MSP7.

### Effects of deletion of *msp7* on histopathology of lungs, liver and spleen in infected rats

**Figure 3:**
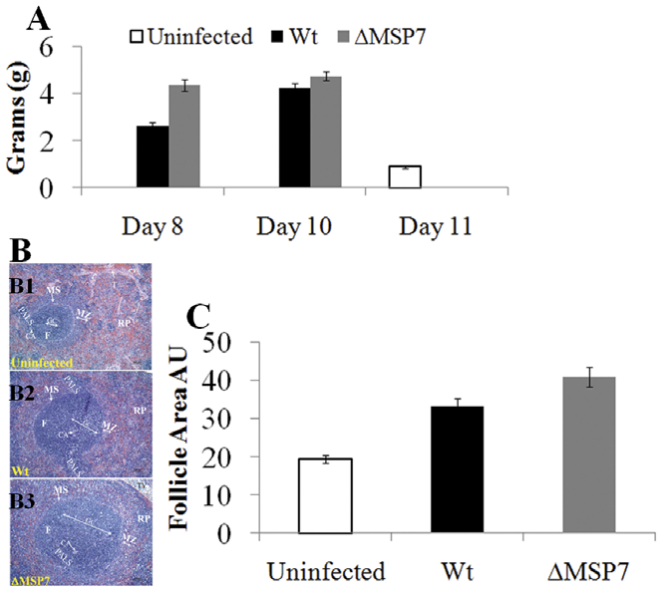
Mass and histological analysis of the rat spleen during infection. (A) Comparison of spleen mass on days 8 and 10 of infection. (B) H&E stained formalin-fixed paraffin embedded spleen sections from uninfected rats and rats infected with *P. berghei* wild type or *Δmsp7* at day 8 p.i. Representative follicles and germinal centers are shown. The greatest size increase is seen in infections with *Δmsp7* (B3). There is disappearance of the marginal zone (MZ) in both wild type and mutant parasite-infected rats (B2&B3). There is infiltration (multiplication) of nucleated cells within the red pulp (RP) (B2&B3). (C) Quantitative analysis of spleen follicle size from uninfected and infected (wild type and *Δmsp7*) rats on day 8 post-infection. For each rat, the size of follicles was analyzed in four different spleen sections. In total, 75 and 81 spleen follicles were analyzed for uninfected rats (n = 4) and rats infected either with wild type (n = 3) or *Δmsp7* mutant (n = 4) parasites, respectively. Scales represent standard error of the means. T, trabecula; RP, red pulp; GC, germinal center; CA, central artery; F, follicle; TV, trabecular vein; MS, marginal sinus; MZ, marginal zone; PALS, periarteriolar lymphoid sheaths. Original magnification, x100.

To better understand the differential effects of wild type and *msp7* mutant infections on the host, we analyzed lungs, liver and spleen on histological sections. Organs were removed at day 8 (peak parasitemia) and day 10 (immediately after the onset of anemia). Although infection resulted in recruitment of immune cells to the liver, there were no major qualitative or quantitative differences in cell infiltrates observed in mutant or wild type infections (data not shown). No differences were seen in the general morphology of the lungs (data not shown). In contrast there was a ∼ two fold increase in spleen weight at day 8 in mutant relative to wild type infection, but this difference was no longer observed by day 10 (Figure 3A). Quantitative analysis of H&E-stained spleen sections taken at day 8 from uninfected and infected animals showed an increase in follicle size in animals infected with mutant parasites relative to wild type (Figure 3B&C). There may also be changes in the germinal centers and infiltration of nucleated cells within the red pulp (RP) (Figure 3B), however additional detailed studies, beyond the scope of the present characterization, are needed to provide molecular and mechanistic analyses of changes in the spleen. Nonetheless our data in Figure 3 support the notion that deletion of *msp7* in the parasite induced dynamic changes in the host spleen.

### Effects of deletion of *msp7* on circulating levels of cytokines in rat plasma

**Figure 4:**
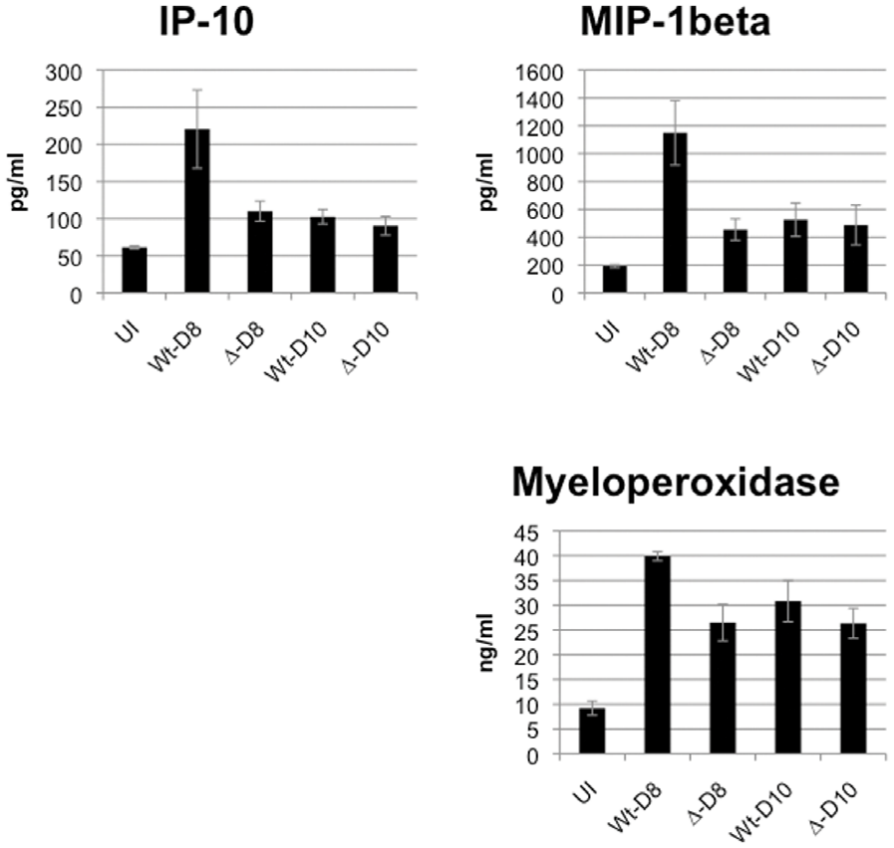
Analysis of rat plasma. Rats were infected and monitored for parasitemia and Hb levels until day 8 or 10 post-infection, at which point they were exsanguinated and plasma was isolated. Interferon-gamma induced protein 10 (IP-10); macrophage inflammatory protein-beta (MIP-1beta) and myeloperoxidase appear to be elevated on Day 8 in wild type-infected animals relative to mutant-infected animals. However, when we adjusted for the number of tests realized, P-values for IP-10, MIP-1beta, and myeloperoxidase all were >0.05. N = 3 wild-type; N = 4 *Δmsp7*.

To further characterize the effects of infection by wild type and *msp7* mutant parasites, we investigated whether there was a change in 59 inflammatory cytokines (Figure S5) detected in plasma on Day 8 (peak parasitemia). Blood plasma levels of IP-10 and MIP-1 beta appeared higher in infection with wild type parasites relative to infection with the *msp7* mutant (Figure 4), with MIP-1 beta displaying a larger wild type to mutant ratio (2.5 fold difference, Figure 4) The third analyte was myeloperoxidase, which is mostly found in neutrophil granulocytes. MIP-1 beta and IP-10 are both chemo-attractants for various immune cells including natural killer cells, monocytes and T-cells. However, P values for these cytokines were greater than 0.05 after we adjusted for the number of tests realized and thus further analysis is needed to conclude on the significance of these data to deletion of *msp7*. No significant differences were seen in these analytes on day 10 (see Fig. S5).

## Discussion

We investigated two rodent models to study the effects of deletion of a single gene on infection and disease. The semi-immune Balb/c mouse model is complicated by the long period of preparation, such that by the time the animals are semi-immune it is difficult to separate effects of age from those of immunity. Both the aged rat and mouse models have been shown to have an end point of death that is likely not merely due to parasite load. Further, the observed death is not a manifestation of other underlying malarial pathology, such as cerebral malaria, since no neurobehavioral changes were evident during infection [29]. In the aged mouse, the parasitemias associated with both wild type and mutant parasite infection are elevated and yet distinct (∼42% and ∼75% respectively) and thus it is difficult to separate the contribution of relatively high parasite burdens to host inflammation.

In the rat, we see a self-resolving infection that peaks at 3% parasitemia for wild type and 2% parasitemia for mutant infections. The aged/adult rat model is an accepted model for malarial anemia, although on average the changes we detected in hemoglobin levels are smaller than previously reported. Nonetheless we find that infection with the *Δmsp7* mutant abrogates death and results in a measureable reduction of anemia. Whether deposition of MSP7 on the surface of uninfected erythrocytes (either directly or indirectly) plays a mechanistic role in this process has yet to be tested. The finding that the deletion of *msp7* abrogates host death was unexpected. The small reduction in hemoglobin levels could have been expected, because malarial anemia is a complex disease pathology and any single antigen may play a small component role. Nonetheless our studies strongly support the potential of the rat model to assess additional parasite proteins that, like MSP7, are not essential for infection but may contribute to anemia and death.

Although circulating parasitemias for both wild type and *Δmsp7* mutants in rats were low, it should be noted that total body parasite biomass has yet to be assessed. It is formally possible that *Δmsp7* mutants may differentially accumulate (this may or may not be the same as classical sequestration) in tissues. This may not necessarily have been seen in histological sections since these were treated to remove circulating blood. Small differences in peripheral parasitemia if sustained over a few days may result in marked differences in cumulative parasite biomass and might influence anemia. Further, where RBCs are being destroyed and there is evident anemia, the absolute number of RBCs per ml of blood also changes thus the absolute number of pRBCs in the circulation may be an important determinant.

Our initial analysis of the host response suggests that deletion of *msp7* results in greater increase in follicle size and changes in germinal centers in the spleen of wild type- infected animals. It remains unclear whether plasma levels of inflammatory cytokines are different in wild type and mutant infected animals. Since the effects on anemia are relatively minor, it is clear that anemia is not the cause of death. The relative increase in follicle size in spleens from animals infected with the mutant parasite, suggest that deletion of *msp7* may alter the B cell response. Further analysis is required to understand changes in immune cell migration to the spleen as well as the immunological component of both hemoglobin reduction and host death.

The *msp7* gene is conserved across *Plasmodium* species. The protein has been investigated as a vaccine candidate because it is located at the surface of the invasive blood stage merozoite where it is in a complex with merozoite surface proteins 1 and 6 in *P.falciparum* [30]. Our data suggest that deletion of *msp7* results in an abrogation of death and a decrease from 3% to 2% in peak, circulating parasitemia in the context of malarial infection. Thus care should be taken when considering this and similar ligands for a vaccine, since its role in disease is not yet fully understood. Our data also indicate that rodent models may be utilized in testing the disease potential of invasion ligands that are under investigation as vaccine candidates.

## Supporting Information

Figure S1 **Analysis of** ***P. berghei msp7*** **knock out by PCR and Southern blotting.** (A). Specifically designed primers allowed for the distinction of *msp7* knock-out and wild type lines of *P. berghei*. (B) Successful integration of plasmid pDHΔMSP7 results in unique PCR products for both wild type parasites with the *msp7*gene and parasites with full integration of the targeting plasmid into the endogenous gene (TOP). Two percent agarose gel of PCR products obtained from reactions using genomic DNA from wild type *P. berghei* ANKA or cloned putative *Δmsp7* parasites (BOTTOM). (C) Southern blot analysis of wild type and *Δmsp7* parasites (both lines; Tewari [MSP7ko] and from this work [ΔMSP7]), digested with *Eco*R1 and *Hind*III, and probed with an *msp7* probe.
(TIF)

Figure S2 **Aged mouse model of death.** (A) Average parasitemia and Hb levels for acute mouse infections in 5– 6 month old mice. (B) Number of mice that survived in the aged mouse model. Each graph is representative of two experiments, N = 20 mice. Hb; hemoglobin
(TIF)

Figure S3 **Acute infection established in mice by i.v. injection of 1000 parasites.** (A) Average parasitemia achieved in acute mouse infections where 5–6 month old mice were injected with 1000 parasites of either wild type (black) or mutant (grey) strains. (B) Days at which 50% death is seen in 6–8 week and 5–6 month mouse models injected with 1000 parasites, i.v. Data combines two experiments, with N = 10 animals in each.
(TIF)

Figure S4 **Analysis of cytokines in mouse plasma.** Mice were infected and monitored for parasitemia until day 7 (wild-type) or 11 (Δ*msp7*) post-infection, at which point they were exsanguinated and plasma isolated. Interferon-gamma (IFN-gamma); interleukin 18 (IL-18); interferon-gamma induced protein 10 (IP-10); monocyte chemoattractant protein 1 and 3 (MCP-1 and MCP-3); macrophage inflammatory protein 2 (MIP-2). N = 5 wild-type; N = 3 Δmsp7.
(TIF)

Figure S5 **Raw data for rat cytokine analysis.** The complete 59 analyte panel of the version 2.0 Rodent MAP profiling from Rules Based Medicine, Inc was tested in plasma drawn on day 8 and day 10 of rats infected with wild type parasites or *Δmsp7* mutants.
(XLS)
