# Supplementary figures and images for: Deletion of a Malaria Invasion Gene Reduces Death and Anemia, in Model Hosts
Source: PLoS One. 2011 Sep 28;6(9):e25477. doi: 10.1371/journal.pone.0025477 (PMC3182240; doi:10.1371/journal.pone.0025477)

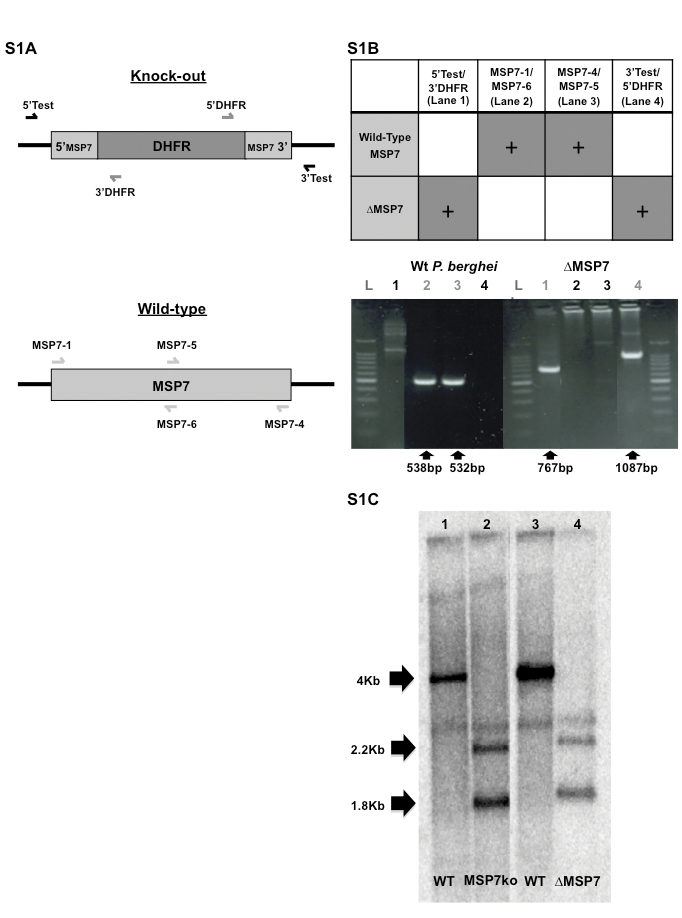

Supplement: Figure S1 — Analysis of P. berghei msp7 knock out by PCR and Southern blotting. (A). Specifically designed primers allowed for the distinction of msp7 knock-out and wild type lines of P. berghei. (B) Successful integration of plasmid pDHΔMSP7 results in unique PCR products for both wild type parasites with the msp7gene and parasites with full integration of the targeting plasmid into the endogenous gene (TOP). Two percent agarose gel of PCR products obtained from reactions using genomic DNA from wild type P. berghei ANKA or cloned putative Δmsp7 parasites (BOTTOM). (C) Southern blot analysis of wild type and Δmsp7 parasites (both lines; Tewari [MSP7ko] and from this work [ΔMSP7]), digested with EcoR1 and HindIII, and probed with an msp7 probe. (TIF) [file pone.0025477.s001.tif]

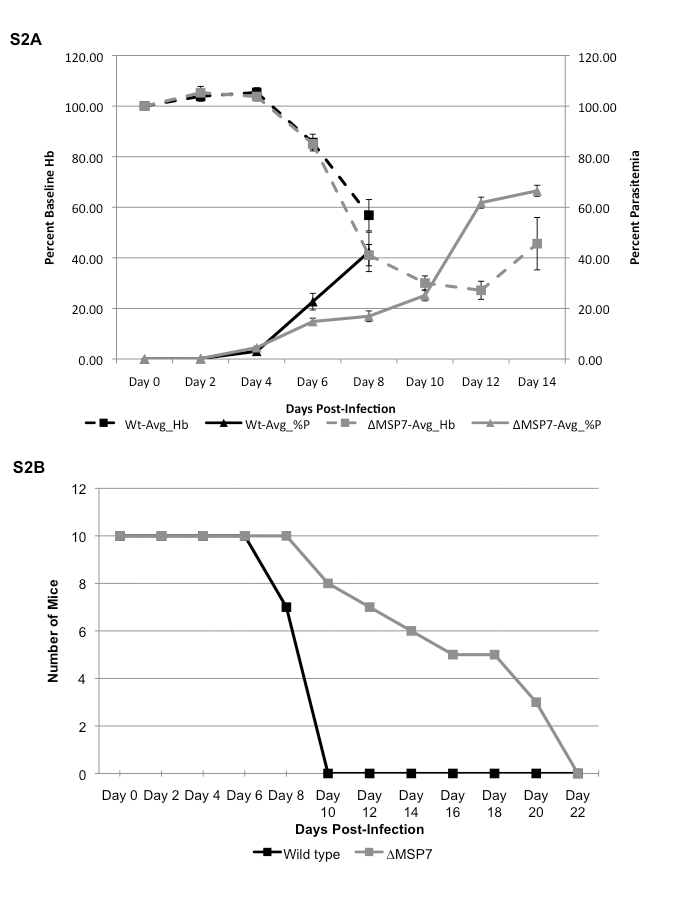

Supplement: Figure S2 — Aged mouse model of death. (A) Average parasitemia and Hb levels for acute mouse infections in 5– 6 month old mice. (B) Number of mice that survived in the aged mouse model. Each graph is representative of two experiments, N = 20 mice. Hb; hemoglobin (TIF) [file pone.0025477.s002.tif]

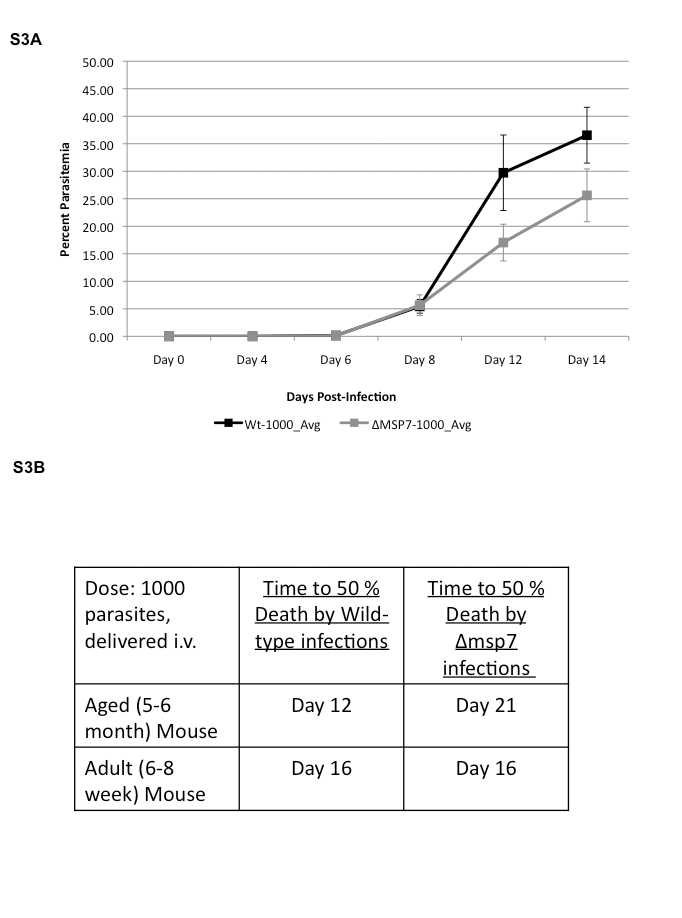

Supplement: Figure S3 — Acute infection established in mice by i.v. injection of 1000 parasites. (A) Average parasitemia achieved in acute mouse infections where 5–6 month old mice were injected with 1000 parasites of either wild type (black) or mutant (grey) strains. (B) Days at which 50% death is seen in 6–8 week and 5–6 month mouse models injected with 1000 parasites, i.v. Data combines two experiments, with N = 10 animals in each. (TIF) [file pone.0025477.s003.tif]

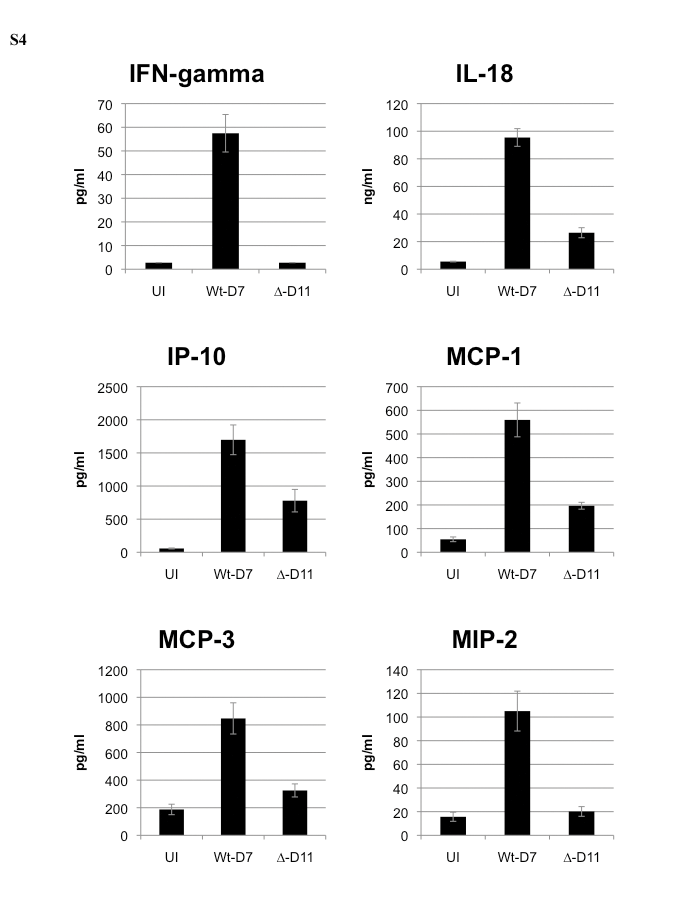

Supplement: Figure S4 — Analysis of cytokines in mouse plasma. Mice were infected and monitored for parasitemia until day 7 (wild-type) or 11 (Δmsp7) post-infection, at which point they were exsanguinated and plasma isolated. Interferon-gamma (IFN-gamma); interleukin 18 (IL-18); interferon-gamma induced protein 10 (IP-10); monocyte chemoattractant protein 1 and 3 (MCP-1 and MCP-3); macrophage inflammatory protein 2 (MIP-2). N = 5 wild-type; N = 3 Δmsp7. (TIF) [file pone.0025477.s004.tif]
